# Staphylococcal proliferation on skin models to investigate novel anti‐infective treatments against dysbiosis

**DOI:** 10.1002/btm2.70124

**Published:** 2026-03-26

**Authors:** Sarah Frisch, Samy Aliyazdi, Jacqueline Rehner, Georges Schmartz, Caroline Gevaerd, Lorenz Latta, Barbara Veldung, Sören L. Becker, Andreas Keller, Ulrich F. Schaefer, Brigitta Loretz, Thomas Vogt, Claus‐Michael Lehr

**Affiliations:** ^1^ Helmholtz‐Institute for Pharmaceutical Research Saarland, Helmholtz Center for Infection Research Saarbrücken Germany; ^2^ Saarland University Saarbrücken Germany; ^3^ Institute of Medical Microbiology and Hygiene, Saarland University Homburg Germany; ^4^ Clinical Bioinformatics, Saarland University Saarbrücken Germany; ^5^ Clinic for Dermatology, Venereology, and Allergology Homburg Germany; ^6^ Specialist in Plastic and Aesthetic Surgery Saarbrücken Germany; ^7^ PharmaScienceHub, Saarland University Saarbrücken Germany

**Keywords:** antibiotic tolerance, bacterial infection, biofilm, hidradenitis suppurativa, in vitro models, microbiome, rhamnolipids

## Abstract

Inflammatory skin conditions like Acne inversa are characterized by dysbiosis, an imbalance of commensal and pathogenic bacteria, posing challenges for specific treatments. Consequently, we investigated how biofilm formation, low‐nutrition skin environments, and air interfaces influence susceptibility to anti‐infective treatments in mixed bacterial cultures. To achieve this in a cost‐effective and reproducible manner, we developed a simplified substrate made of gelatin, hyaluronic acid, chondroitin sulphate, and alginate (=Gel‐Alg). This in vitro model simulates biofilm cultivation on skin surfaces for aerobic bacteria. We selected *Staphylococcus aureus* and *Staphylococcus epidermidis* as two clinically relevant strains, which are also abundant in Acne inversa. We tested single and mixed cultures under different conditions: (i) nutrient broth, (ii) Gel‐Alg substrate, (iii) EpiDerm™ commercial skin model, and (iv) ex vivo human skin. Proliferation, measured by colony‐forming units, was comparable across most conditions, except for human skin. Metabolic activity, assessed via Presto Blue staining, revealed significant differences. Dual‐species cultivation and quantification by viability PMA qPCR indicated dominance of *S. epidermidis* over *S. aureus* in skin‐like environments. Treatments with biofilm‐dissolving rhamnolipids, the antibiotic vancomycin, and combinations thereof demonstrated varying efficacy in single and mixed cultures. While the drug combination could almost completely eradicate staphylococcal biofilms in broth, susceptibility varied in skin‐like models and moreover strongly depended on temperature (37°C vs. 32°C). In conclusion, this study suggests that reductionistic models, while mimicking key features, could be valuable for early selective antimicrobial drug development for specific applications like Acne inversa therapy.

**Translational Impact Statement**

This study highlights the development of a novel in vitro model that mimics important basic aspects of bacterial cultivation on skin, such as air interface and minimal nutrition, enabling the investigation of bacterial interactions and response to treatments. By revealing how environmental factors influence bacterial susceptibility to antimicrobial therapies, the work provides essential insights that may improve treatment strategies for inflammatory skin conditions like Acne inversa. Ultimately, these findings could facilitate the effective development of targeted therapies and enhance patient outcomes in managing these challenging conditions.

## 1 INTRODUCTION

Acne inversa, also known as hidradenitis suppurativa, is a chronic and inflammatory skin condition that manifests in painful, suppurating lesions, primarily affecting the axillary and inguinal regions. The underlying causes are multifactorial and involve bacterial colonization resulting in a so‐called dysbiosis.^1^, ^2^ The latter is characterized by an imbalance of healthy microbiota with a prevalence of pathogenic bacteria over commensals.^3^ This shift can contribute to the development and progression of the disease.^4^ While studies on the altered bacterial abundance in Acne inversa are on‐going,^5^ the microbial composition and its changes remain largely unexplored. The involvement of bacteria in the early stages of disease, characterized by the absence of a protective microbiota biofilm or dysbiosis, is a well‐established aspect of medical understanding. Nonetheless, it remains a pivotal question whether it is the primary disease trigger or consequence. Furthermore, observations of bacterial biofilms within sinus tracts in later and more severe stages of the disease suggest their role as opportunistic colonizers, establishing themselves in the niche subsequent to the rupture of pilosebaceous units.^6^, ^7^

Current therapy options for Acne inversa and associated infections are limited. Treatment with antibiotics like rifampicin and clindamycin is common,^8^ but their use can lead to several disadvantages. These include detrimental effects on beneficial commensal bacteria, an increased risk of antibiotic resistance, and limited efficacy against bacterial biofilms.^9^

The persistent and chronic nature of infections in Acne inversa is due to the deregulation of innate immune responses and initial alterations in the Acne inversa microbiome. Antibacterial therapy may play a crucial role in preventing the progression of early‐stage Acne inversa, as these factors significantly contribute to the formation of biofilms, which pose a considerable challenge to effective treatment.^10^ In biofilms, bacterial aggregates are embedded in a self‐produced matrix of extracellular polymeric substances (EPSs) that protects the cells and reduces drug diffusion.^11^ Moreover, the altered microenvironment characterized by lower nutrients, oxygen, and pH leads to decreased metabolism and formation of dormant or persister cells, which exhibit high antibiotic tolerance.^12^

The development of novel therapeutics against bacterial infections and biofilms is consequently an on‐going research topic: innovative drug delivery systems help to overcome the biofilm barrier, but also the combination of antibiotics with quorum sensing inhibitors or surfactants are promising approaches.^13^, ^14^, ^15^ One example for the latter is rhamnolipids (RLs), some glycolipids produced by *Pseudomonas aeruginosa*, which disrupt the EPS matrix.^16^ These bio‐surfactants have the potential to enhance antibiotic diffusion and thus serve as suitable candidates for efficient co‐treatment against biofilms as shown by Radlinski et al.^17^, ^18^ However, the efficacy in the context of severe skin diseases such as Acne inversa has not yet been explored, highlighting the need for further research in this area.

The skin presents a quite harsh habitat for microorganisms due to its dry and low‐nutrient surface, and reduced temperature of 32°C.^19^ Still, these factors, along with the presence of commensal bacteria during an infection, are often not considered when testing novel anti‐infectives in vitro. Standard procedures to assay antimicrobials typically involve determining the minimal inhibitory concentration (MIC) for planktonic bacteria or the minimal biofilm eradicating concentration for bacterial biofilms.^20^ These methods are easy, inexpensive, and provide information on the potency of new anti‐microbial compounds. However, cultivation in nutrient broth clearly misses some key parameters like air‐interface cultivation mimicking the solid substrate nutrition supply. A model allowing standardization and meeting such cultivation conditions is of particular interest when investigating mechanistic effects or development of sophisticated anti‐infectives therapies (e.g., delivery systems or drug combinations targeting biofilm).

The need for suitable in vitro testing platforms has increased with the prohibition of animal testing for cosmetics (regulation [EC] No. 1223/2009 on cosmetic agents), which led to the development of more advanced skin models. Nowadays, these reach from cell‐based reconstructed human skin to ex vivo samples, with animal (especially porcine) skin also being a well‐established alternative.^21^, ^22^ Such models provide a more physiologically relevant testing environment and are optimized for the growth of human cells and tissue formation. Nevertheless, their complexity comes with high costs and increased variability, which presents certain drawbacks for high‐throughput assays. An alternative can be the use of cultivated cell monolayers^23^ or stratum corneum‐based substrates,^24^ but the existing models are currently still lacking air‐interface or include ex vivo materials.

To address these limitations, we have developed a rather simplified substrate based on gelatin (Gel), hyaluronic acid (HA), chondroitin sulphate (CS), and Alginate (Alg) (=Gel‐Alg). This hydrogel is based on a scaffold originally developed by Quan et al.^25^ but we have extended and optimized its composition by adding alginate to improve stability and to achieve mild cross‐linking with calcium chloride. While this model does not represent a physiologically relevant scaffold, it allows us to mimic important basic aspects of bacterial cultivation on skin, such as air interface and minimal nutrition. Simultaneously, this model offers affordability and reproducibility, which are advantageous for the early‐stage testing of novel drug candidates. Subsequently, optimized anti‐infectives can undergo a more comprehensive analysis using complex models, like for instance reconstructed human epidermis (RHE). Moreover, the viscoelastic properties of the substrate enable a degree of adaptability within the context of 3D (bio‐)printing.

In our study, we aimed to find out if such a simplistic experimental set‐up can be used for the cultivation of two bacterial species and testing those against selective treatments. These experiments are listed in Part A of the Results and Discussion section. Additionally, we conducted a small, controlled case study, comparing the microbiome of Acne inversa‐affected body sites to healthy regions (forehead or arm) from the same individuals (*n* = 18) to provide some additional data on changes in bacterial composition (Part B).

**FIGURE 1:**
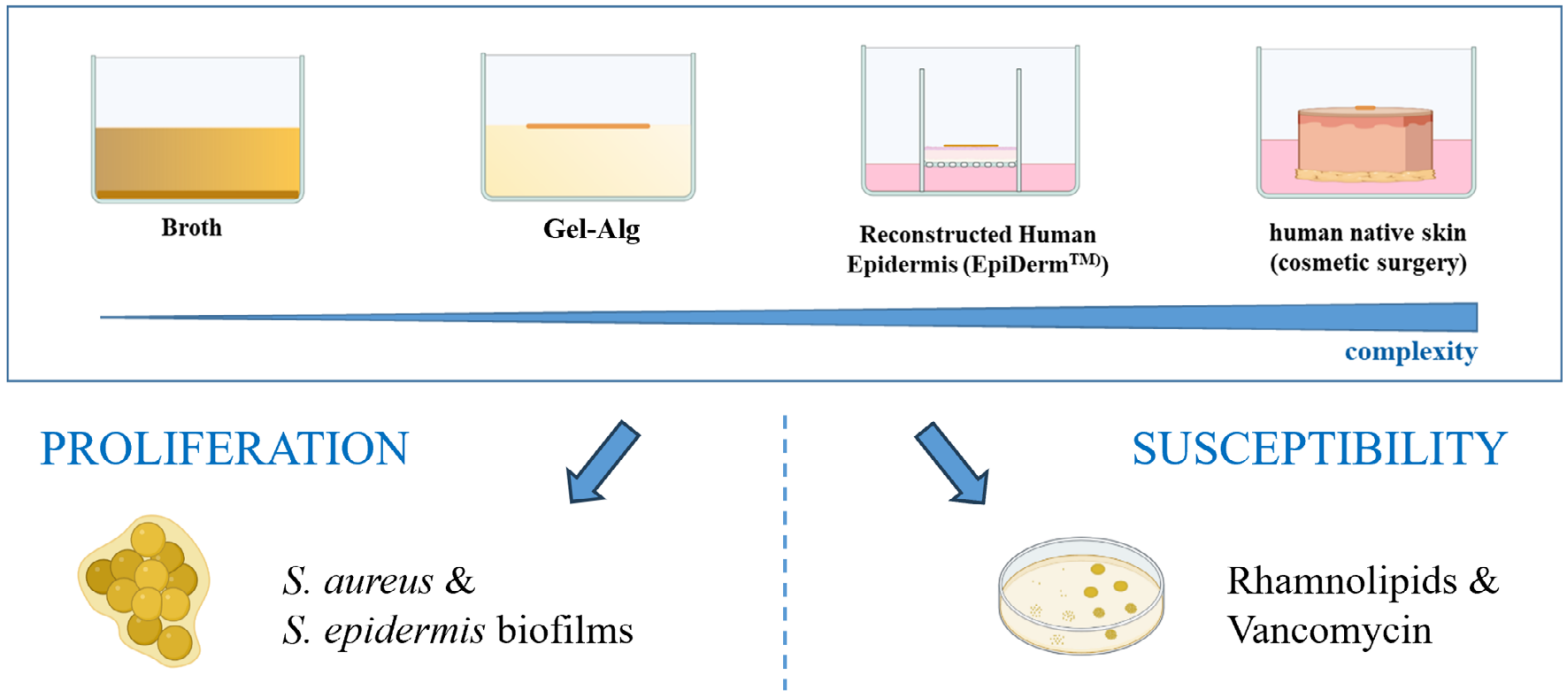
Overview of tested cultivation conditions for *Staphylococcus aureus* and *Staphylococcus epidermidis* biofilm proliferation, from left to right: broth (liquid culture, standard condition), minimal air interface model Gel‐Alg, commercial reconstructed human epidermis (RHE, Epi‐Derm™, MatTek), and human native skin, obtained from cosmetic surgery. In addition, the models were examined for the impact on the effectiveness of co‐treatment with rhamnolipids and vancomycin.

For the proliferation and susceptibility testing (Part A), we focused on *Staphylococcus aureus* and *Staphylococcus epidermidis* as they are commonly associated with skin infections,^26^, ^27^ and also found abundant at the infection site of Acne inversa patients.^5^ Bacterial quantification in dual‐bacterial cultures was performed via viability qPCR. We incorporated relevant controls such as (i) nutrient broth, (ii) a commercial reconstructed skin model (RHE, EpiDerm™, MatTek), and (iii) ex vivo skin from cosmetic surgery. These models, as well as cultivation of two strains as bacterial co‐culture, enabled a more comprehensive analysis of factors affecting proliferation and susceptibility toward antibiotics (Figure 1). Treatment efficacy in different conditions was finally explored via a combination of RLs and vancomycin, as this experimental combination showed more promising and selective results than previously tested antibiotics such as rifampicin (data not shown). In the context of specific applications like bacterial dysbiosis in Acne inversa, we hypothesize that utilizing relevant in vitro testing platforms and appropriate cultivation conditions might alter the treatment response when investigating novel anti‐infectives.

## 2 RESULTS AND DISCUSSION

### 2.1 Part A: Bacterial proliferation and susceptibity in skin models

#### 2.1.1 Staphylococcal proliferation in different skin models

**FIGURE 2:**
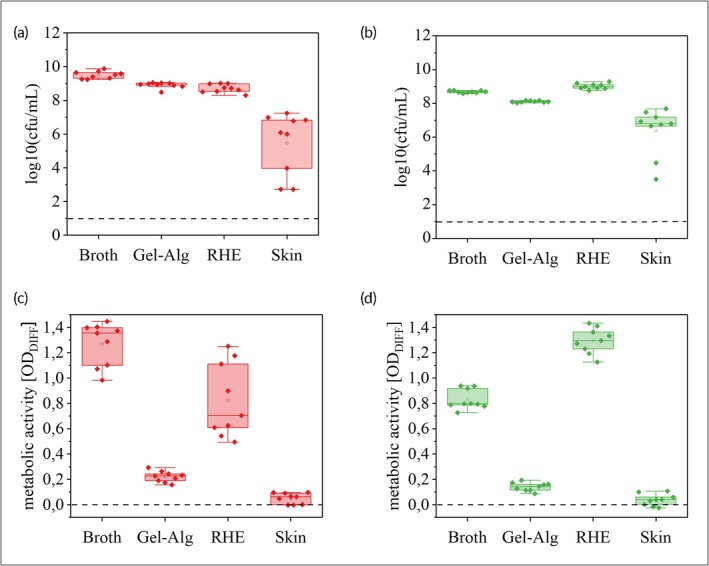
Growth and metabolic activity in different conditions: broth, skin substrate Gel‐Alg, reconstructed human epidermis (RHE, EpiDerm™, MatTek) and ex vivo skin. Growth via log10(cfu/mL) of *Staphylococcus aureus* (a) and *Staphylococcus epidermidis* (b) single biofilms. One‐way ANOVA revealed significant difference of ex vivo skin to all other conditions tested. Metabolic activity via Presto Blue staining and OD_DIFF_ of *S. aureus* (c) and *S. epidermidis* (d) single biofilms. All conditions showed statistically significant difference as tested via one‐way ANOVA. Significance levels are only visualized via asterisks (*) in Figure S1. *N* = 3 experiments with *n* = 9 samples.

Four different conditions were compared regarding bacterial growth and metabolic activity as depicted in Figure 2: broth, Gel‐Alg, RHE, and skin. In general, it was observed that variations were higher for metabolic activity than bacterial count for both strains, *S. aureus* and *S. epidermidis*.

Growth data is depicted in Figure 2A for *S. aureus* and Figure 2B for *S. epidermidis*. For cultures in nutrient broth, high proliferation of around 9 log_10_(cfu/mL) was reached with little standard deviation, which can be expected due to standardization. Gel‐Alg also demonstrated good reproducibility, but the bacterial count was slightly lower for both species. RHE samples varied to a greater extent but were in a comparable range, being slightly lower for *S. aureus* and higher for *S. epidermidis*, respectively. Only skin appeared to be an outlier as growth is strongly reduced, and values scattered to a higher extent (>3 log_10_(cfu/mL)). This might be ascribed to the ex vivo characteristic, including intra but also inter patient variance. An alteration in growth and proliferation dependent on nutrition was already proven in several publications.^28^, ^29^, ^30^ However, our set‐up reaching from standardized broth to ex vivo skin could furthermore provide some valuable insights on other factors like air interface and nutrition.

A different trend was observed for metabolic activity (Figure 2C for *S. aureus* and Figure 2D for *S. epidermidis*) with all conditions showing statistically significant differences (see also Figure S1). OD_DIFF_ values for broth cultures are high for both strains with around 1.2 and 0.85 for *S. aureus* and *S. epidermidis*, respectively. The liquid‐covered condition paired with high nutrients seems to favor biochemical activity. All other systems were at air interface, which might have affected the bacterial metabolism regarding the strong reduction, especially for Gel‐Alg and skin. However, RHE stands out with a much higher value. These might be related to a “richer” cell medium in the basolateral compartment of the model. RHE is quite permeable with low diffusion resistance, so that fluids from the basolateral department can leak upward.^31^

#### 2.1.2 Bacterial co‐cultures

**FIGURE 3:**
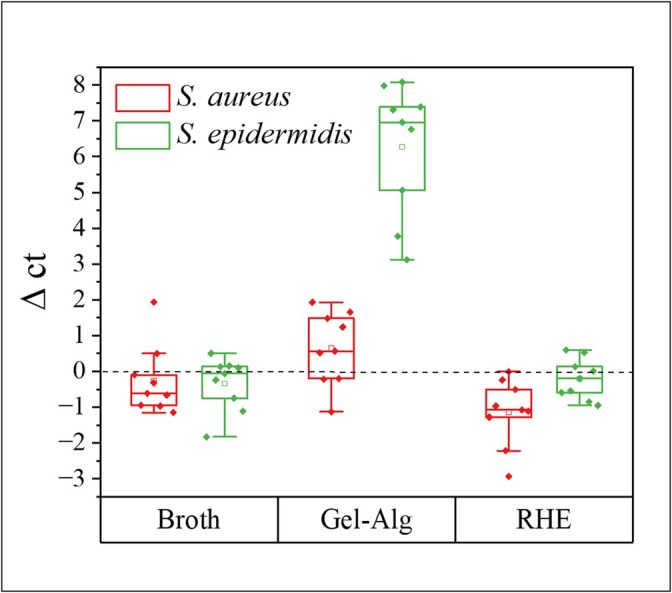
Quantification by viability qPCR following mixed cultivation of *Staphylococcus aureus* and *Staphylococcus epidermidis* in different conditions: broth, skin substrate Gel‐Alg and reconstructed human epidermis (RHE, EpiDerm™, MatTek), depicted as Δct between single and mixed cultures. *N* = 3 experiments with *n* = 9 samples.

The proliferation of *S. aureus* and *S. epidermidis* was further characterized in three models. Human skin was excluded here as a consequence of more distinct variations. Starting with a 1:1 ratio of both staphylococci, alterations in their abundance after a 24 h growth in co‐cultivation in the respective model were determined using a viability PCR. It uses PMA treatment to distinguish dead and living cells when quantifying via qPCR. The results are shown in Δct as fold‐change between single and dual‐bacterial culture (Figure 3). Here, a Δct of 3.3 corresponds to 1 log difference in cfu/mL. For cultivation in broth, the changes were rather marginal and within the range of 1 log difference. When using Gel‐Alg, however, a clear prevalence can be observed with enhanced growth of *S. epidermidis* and a mean Δct of 6.7. This might be related to better adaptability to skin‐like conditions as a major skin commensal microbe. Although this trend was not confirmed with the RHE, *S. epidermidis* still appeared more dominant in this environment due to the reduced bacterial count of *S. aureus*.

Fredheim et al. showed that growth of both staphylococci was not inhibited in standard cultures, confirming our findings in nutrient broth. However, the authors also observed that the presence of *S. epidermidis* could affect *S. aureus* biofilm formation, which might explain the differences in altered (skin) conditions.^32^ Van der Krieken et al. cultivated both staphylococcal species on a model based on human SC/callus suspension. Interestingly, in their set‐up, a prevalence of *S. aureus* was observed after 24 h cultivation, but it needs to be mentioned that nutritional differences and the additional presence of *P. acnes* might lead to substantial changes affecting bacterial crosstalk and proliferation.^24^ Jordana‐Lluch et al. found comparable cfu values in a *S. aureus* and *S. epidermidis* dual species biofilm with slight but not significant increase of *S. epidermidis* when cultivated in the presence of human keratinocytes (HaCaT cells).^23^ In a study of Kohda et al., the authors could also show comparable results using a 3D epidermis equivalent with *S. aureus* being significantly lower in the co‐culture with *S. epidermidis* than in *S. aureus* monoculture.^33^ These results show that our simplified model at least in the tested setting seems to have comparable predictivity when testing for staphylococcal interaction in a skin‐like environment despite not covering all aspects like the presence of cells. However, this needs to be confirmed for other relevant strains and bacterial species, which is the subject of ongoing research.

#### 2.1.3 Rhamnolipid efficacy

**FIGURE 4:**
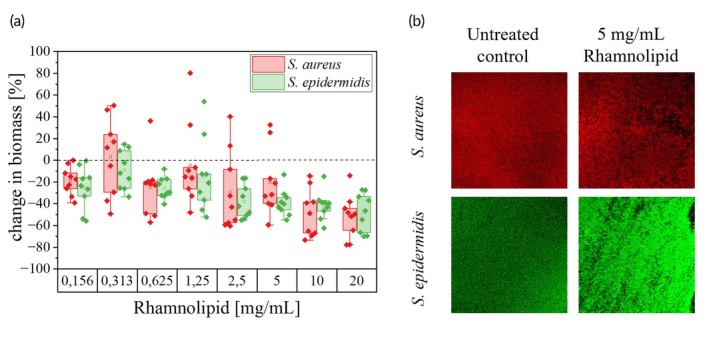
Rhamnolipid (RL) efficacy for *Staphylococcus aureus* and *Staphylococcus epidermidis* single biofilms in broth. (a) Relative biomass in biomass (%) via crystal violet assay after 2 h RL treatment. *N* = 3 experiments with *n* = 9 samples. (b) Exemplary images of untreated and RL treated biofilms after EPS matrix staining with Film Tracer SYPRO ruby.

To test RL efficacy about the EPS reduction a crystal violet assay was performed with different RL concentrations (Figure 4a). Here, biomass was reduced with increasing RL concentration up to ca. 50% for both tested strains. Some differences were noted, particularly at lower concentrations, which may be attributed to changes in metabolic rates or variations in cellular responses to reduced RL levels. Crystal violet staining is commonly used for biomass quantification; however, the processes involved—such as fixation, staining, and multiple washing steps—can introduce variability, also in untreated control samples.

To complement and strengthen these data, images were taken after SYPRO ruby film tracer staining (Figure 4b). EPS disruption was observable in comparison to PBS treated sample at a concentration of 5 mg/mL. Here, no substantial distinction was detected between *S. aureus* and *S. epidermidis*.

RL have already been shown to act on the *S. aureus* cell membrane, potentially the peptidoglycan.^34^ This could explain their inhibitory but also disruptive characteristics.^35^ RLs were tested against *S. aureus* in the context of food borne disease.^16^ Here, the authors reported a biomass reduction of up to 25% in comparable cultivation conditions. Since a similar quantification method was used, this lower effect might be based on different strain and medium. In richer medium (skim milk), a decrease of up to 80% could be achieved. Buonocore et al. explored the RL effect on various levels including biofilm inhibition, biofilm penetration and biofilm degradation.^36^ The latter with comparable success to our results with up to 50% degradation. For *S. epidermidis*, such assays have not yet been performed but Sodagari et al. could demonstrate a reduced attachment of the strain on glass upon treatment with RLs.^37^

Overall, the efficacy of RL in our set‐up was confirmed and allied with published results. Furthermore, we could extend this and reveal comparable effects of RL treatment on laboratory biofilm‐forming strains of both *S. epidermidis* and *S. aureus*.

#### 2.1.4 Co‐treatment of rhamnolipids and vancomycin

**FIGURE 5:**
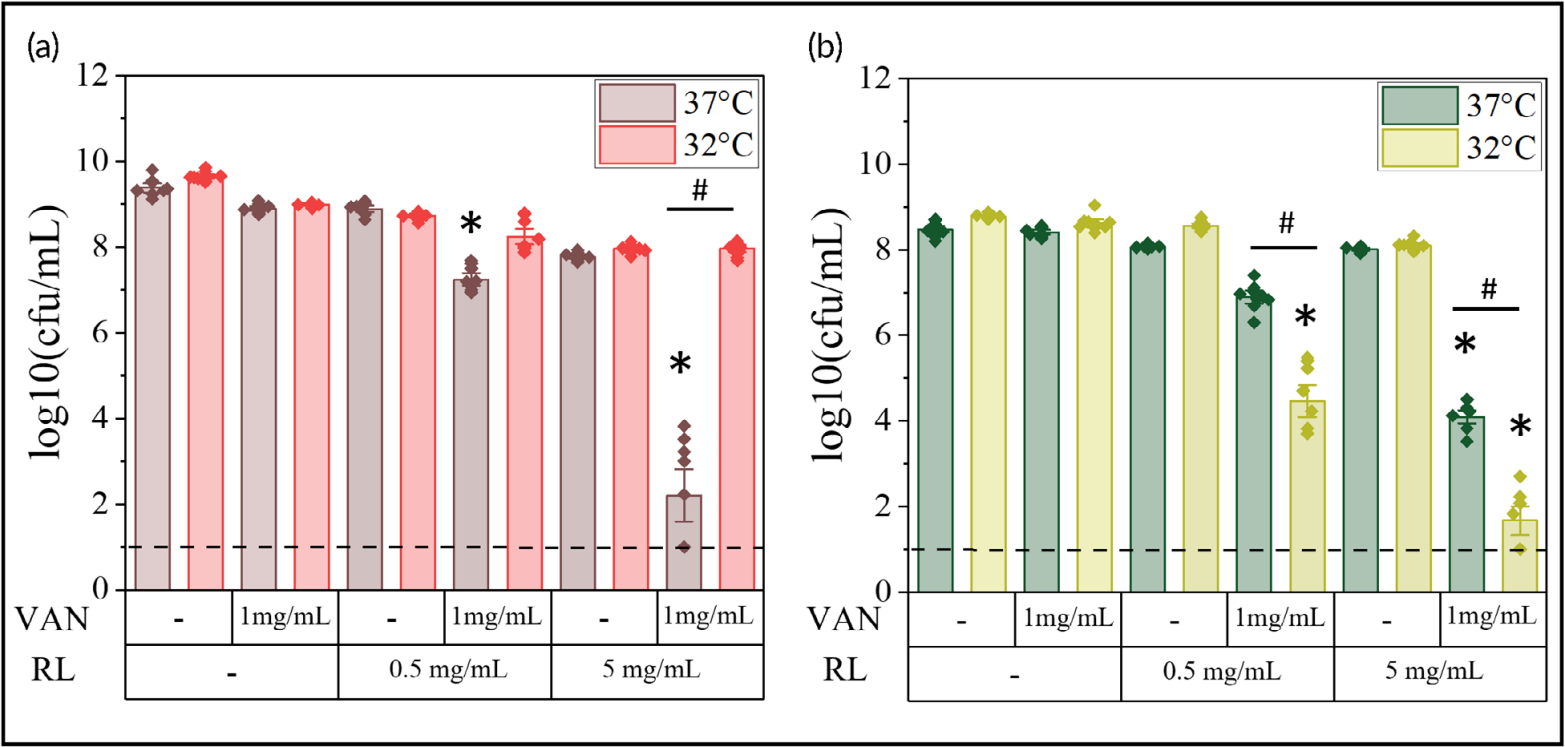
Rhamnolipid (no ‐,0.5 and 5 mg/mL, respectively) and Vancomycin (no ‐, and 1 mg/mL) efficacy via log10(cfu/mL) at 37°C and 32°C for *Staphylococcus aureus* (a) and *Staphylococcus epidermidis* (b) single biofilms. All differences were statistically significant at *p* < 0.05. Here, we highlighted only effects with Δlog10(cfu/mL) >2; asterisks (*) referring to different treatments at the same temperature and hashes (#) indicating effects of different temperatures for the same treatment. Full results for statistical analysis are shown in Tables S2 and S3. (2‐way‐ANOVA or paired *t* test, respectively, *N* = 3 experiments with *n* = 9 samples).

In our study, vancomycin was chosen as a model antibiotic which might profit from the reduced EPS barrier due to its relatively big size (1.45 kDa) and mechanism of action (inhibition of cell wall synthesis). Hence, we tested different combinations of RL and vancomycin concentrations—first in broth only—for both bacterial species. Additionally, we investigated the temperature effect by including a 32°C condition. The results are shown as log_10_(cfu/mL) in Figure 5a for *S. aureus* and Figure 5b for *S. epidermidis*.

Single treatment with vancomycin as well as low (0.5 mg/mL) or high (5.0 mg/mL) RL concentration resulted in a minor reduction of bacterial count. As shown in Tabel S2, the differences were in most cases statistically significant towards the untreated control but amounted <2 log_10_(cfu/mL) With a combination of low RL and vancomycin, the efficacy was only slightly enhanced. However, for both bacteria, a synergistic effect could be seen at 37°C with the combination of high RL concentration and vancomycin. Here, a remarkable 4.4 and 7.9 log reduction was achieved for *S. epidermidis* and *S. aureus*, respectively.

The combination of biosurfactant and antibiotic has already been shown to be a promising approach.^38^, ^39^ Furthermore, RL was already successfully used to treat enterococci infection when combined with linezolid.^40^ Radlinski et al. could also observe a potentiation of vancomycin killing of *S. aureus* when *P. aeruginosa* exoproducts—containing RL among others—were added.^17^ The study did not correlate these results to RL activity on its own, but the data supports our findings regarding some synergistic effects of both drug candidates.

In addition to treatment combination, we investigated the effect at lower skin temperature (32°C). Table S3 depicts Δlog_10_(cfu/mL) for temperature in each treatment group, including statistical significance. Single treatment with RL or vancomycin was comparable to that at 37°C. Despite showing some significant differences, Δlog_10_(cfu/mL) is <0.3 log_10_(cfu/mL) for these treatment groups. However, when considering the combination of high RL concentration and vancomycin, some distinctions were revealed. While the treatment efficiency was even higher for *S. epidermidis*, reaching almost full eradication (7.1 log reduction and Δlog_10_(cfu/mL of 2.4)), it was much more diminished for *S. aureus*. (1.7 log reduction and Δlog_10_(cfu/mL of 6.5)). Hence, we conclude that the change in temperature has a high impact on the anti‐infective efficacy.

Silva et al. have also tested the effect of temperature for RL efficacy but compared 37°C to RT and 5°C.^16^ Although our temperature difference was less pronounced, we can conclude from both findings that RL efficacy appears to be temperature dependent. The thermal response of *S. aureus* and *S. epidermidis* against vancomycin has also been tested by others but using a different temperature gap of 37°C and 45°C.^41^ Interestingly, in this scenario, *S. epidermidis* reacted stronger upon this distinction in temperature. On the other hand, Hajdu et al. demonstrated that increased temperature is an enhancement for anti‐infective and also vancomycin efficacy in staphylococcal biofilms.^42^ These findings support the need to consider the effect of temperature when evaluating novel therapy options. This becomes especially important in the context of non‐body temperature regions such as the skin surface and respective local infections.

#### 2.1.5 Susceptibility in skin models and bacterial co‐cultures

To extend the data set, two further experiments were conducted to compare the effect of co‐treatment in different models as well as bacterial co‐cultures. Here, experiments were performed only at 37°C.

**FIGURE 6:**
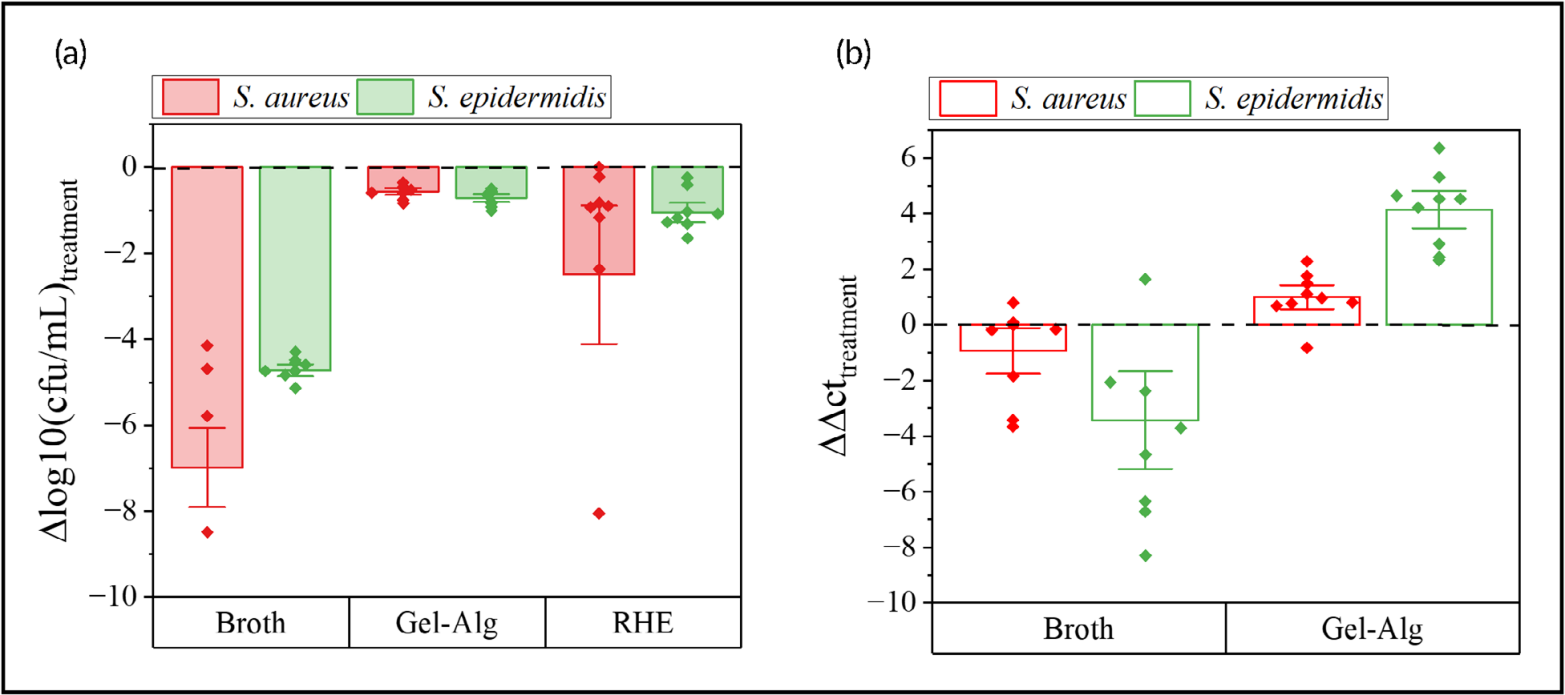
(a) Rhamnolipid + Vancomycin efficacy via Δlog10(cfu/mL)_treatment_ (=cfu_treated_ − cfu_control_) for *Staphylococcus aureus* and *Staphylococcus epidermidis* single biofilms in different conditions. (b) ΔΔct_treatment_ for mixed bacterial culture in broth and on skin substrate Gel‐Alg. First the difference of untreated and treated samples was calculated (Δct_treatment_ = ct_treated_ − ct_untreated_). ΔΔct_treatment_ is then defined as the difference of Δct_treatment_ in single and mixed cultures (ΔΔct_treatment_ = Δct_treatment‐single_ − Δct_treatment‐mixed_). Here, a negative value corresponds to lower efficacy in co‐culture while positive values are associated with a higher one and consequently fewer bacterial survival rates. *N* = 3 experiments with *n* = 9 samples.

First, the model similarity of Δlog_10_(cfu/mL)_treatment_ was noted between broth, Gel‐Alg and RHE (Figure 6a). The strong reduction of bacterial count in broth could not be achieved using the other two models. For Ge‐Alg, less than one log was detected for both species. The treatment was more effective on RHE, especially for *S. aureus* with a reduction of ca. 2.5 Δlog_10_(cfu/mL). For *S. epidermidis* on RHE, the diminution only amounted to an average of 1.5 Δlog_10_(cfu/mL). Again, the medium composition might influence this outcome. Palyczny et al. showed for instance that the MIC of vancomycin against *S. aureus* is much higher in standard cell medium (Dulbeccos Modified Eagle Medium, DMEM) than broth used for bacterial cultivation (tryptic soy broth, TSB), which might correspond to the here observed decreased treatment efficacy in RHE compared to broth (brain heart infusion [BHI]).^43^ For Ge‐Alg, such data is not available yet, but we hypothesize that the minimal supply with nutrients favors the formation of highly tolerant persister cells.

The treatment efficacy analysis in bacterial co‐cultures was limited to broth and Gel‐Alg. Results are displayed in ΔΔct_treatment_ as shown in Figure 6b to display the difference between qPCR ct values of treated single and mixed cultures. As shown by previous experiments analyzing growth in mixed cultivation of both strains, some alterations have been detected, especially for our Gel‐Alg substrate. For susceptibility testing it could be shown that the antibacterial effect upon treatment is not coincident for single and mixed cultures. In broth single cultures, *S. aureus* was more susceptible than *S. epidermidis*. Despite not finding strong growth differences in mixed co‐cultures, *S. epidermidis* was even less susceptible under these conditions.

When single cultures were cultivated on Gel‐Alg, both strains were less inhibited upon treatment. But once adding the RL + VAN combination in the mixed cultures—where an enhanced growth of *S. epidermidis* had been observed—the treatment response also deviated with a much stronger reduction of bacterial count for *S. epidermidis*.

In literature, rather the combination of *S. aureus* and *P. aeruginosa* is used than the one tested in this study. Still, an altered antibacterial effect could be observed in dual species cultures in comparison to single species. Famaei Pirlar et al. suggest that this difference is linked to mutually beneficial interplay among species.^44^ Comparable results for an in vitro mixed culture of *S. aureus* and *P. aeruginosa* were reported by DeLeon et al. who showed that the antibiotic tolerance threshold was higher in co‐cultures.^45^

### 2.2 Part B: Metagenomic analysis of Acne inversa smears

#### 2.2.1 Bacterial abundance in acne inversa smears

**FIGURE 7:**
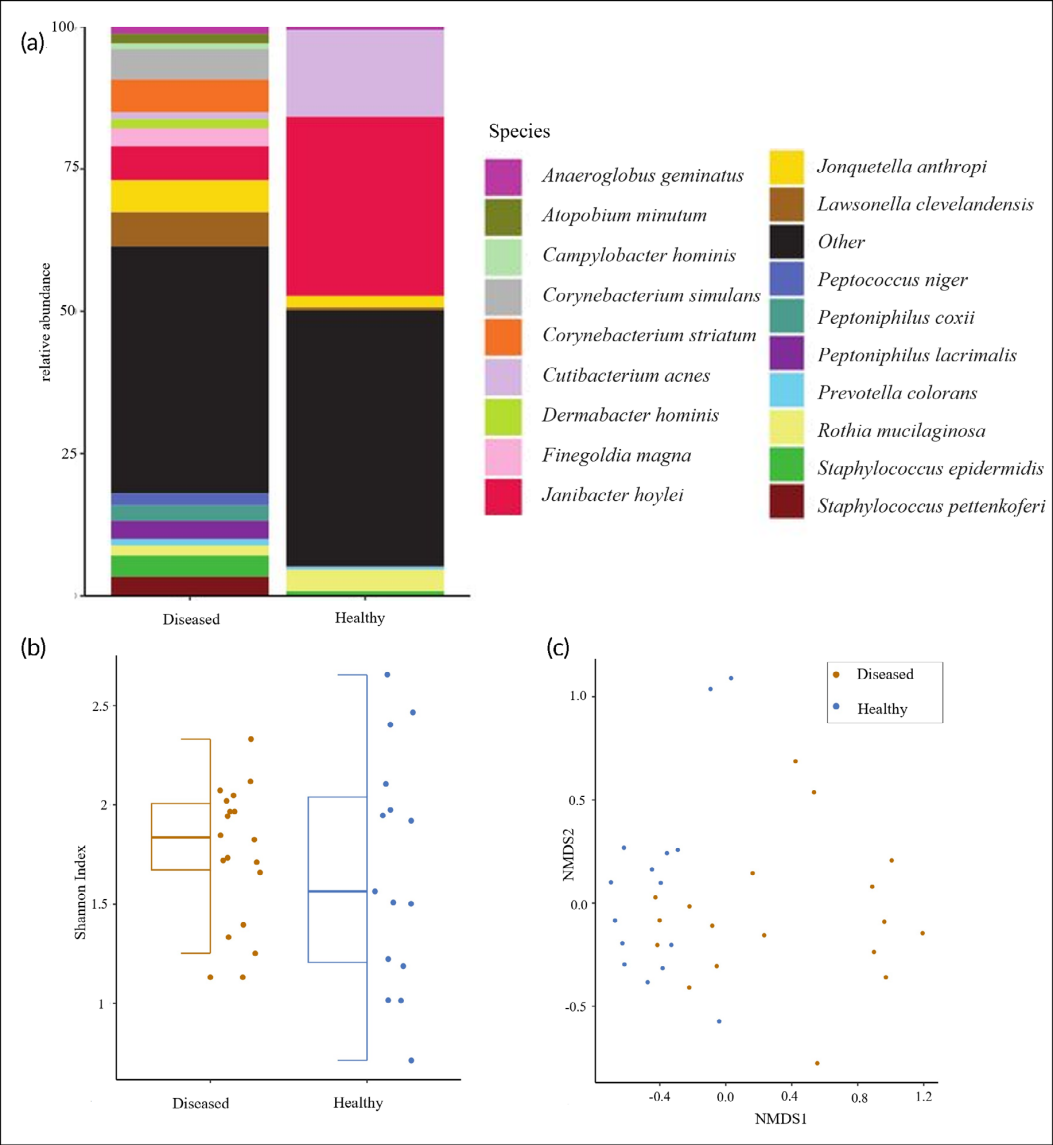
Clinical samples of Acne inversa patients. Bacterial DNA was extracted and metagenomic analysis was performed. Graph (a) shows the distribution of bacterial species, comparing smears from diseased body regions and healthy counterparts. In (b) the alpha diversity is depicted via Shannon index, and (c) demonstrates a distance matrix for beta diversity. *N* = 18 patients.

To explore the microbiome of Acne inversa patients, we included an analysis of smears from diseased body regions. These were compared to smears from unaffected body regions (forehead or arm) of the same patients. A metagenomic analysis revealed that the abundance of species differs strongly between affected and unaffected body regions (Figure 7a). The graph depicts the 18 most abundant species, including for instance corynebacteria, prevotella and staphylococci among others. Regarding the focus of our study, staphylococci, it was for instance observed that *S. epidermidis* is more abundant in diseased smears than healthy counterparts. Despite *S. epidermidis* being rather associated with a healthy skin microbiome, the strain can also contribute to skin diseases as an opportunistic pathogen.^46^ Our second representative, *S. aureus*, was one of the most detected bacteria in that list in the first phase of our skin microbiota analysis (data not shown). Given its association with antibiotic resistance, we kept it as a representative pathogen in our research, even though its abundance dropped with our latest enlargement of the study group. In general, such observation should, however, be taken with care due to the difficulties with sample extraction^47^ and the low number of samples (*N* = 18).

So far, most studies have focused on bacterial species associated with Acne inversa. A study with 50 patients from 2018 also showed a high abundance of *S. aureus* and *S. epidermidis*, as well as *Enterococcus facalis*, *Escherichia coli*, and *Prevotella bivia*.^5^ A french study from 2014 riveted on anaerobic bacteria only and confirmed association of the disease with prevotella but also anaerococcus.^2^ In 2020, Benzecry et al. confirmed most of these findings and also found *Proteus mirabilis* to be highly abundant in Acne inversa smears.^48^ Despite high variations, it can therefore be stated that staphylococci are among the most common species to occur in Acne inversa.

To expand the data set on the analyzed Ance inversa smears, we also investigated alpha diversity, the diversity within one sample, via Shannon index (Figure 7b). Despite not showing a significant difference, a tendency towards higher diversity in healthy samples was detected. This finding is consistent with literature indicating that greater microbial diversity is observed in healthy microbiota across various skin conditions.^49^ However, a significant deviation of the distance matrix for beta diversity, hence the diversity between different samples, could be shown (Figure 7c). Here, healthy samples are rather clustered, meaning that there is higher similarity between the samples. Samples from diseased body parts are rather scattered pointing towards higher variance. In general, our results go along with the hypothesis of diseases being linked to dysbiosis and an alteration of bacterial composition.^3^

## 3 CONCLUSIONS

This study evaluated four in vitro models of different physiological complexity to investigate the effect of anti‐infective treatment against *S. aureus* and *S. epidermidis*, either in single or mixed cultures (Part A). Both staphylococcus strains showed comparable growth in different models in single cultures, but in mixed cultures, a slight superiority of the skin commensal *S. epidermidis* in models with air‐interface (Gel‐Alg and RHE) was observed. The efficacy of biofilm‐dissolving RLs also depended on culturing conditions, being most pronounced in broth in comparison to any of the skin models.

Comparing the viability of two related bacterial species, such as *S. aureus* and *S. epidermidis*, is not trivial and was possible by means of PMA qPCR. We observed substantial changes in antimicrobial efficacy of vancomycin either alone or in combination with RLs. This combination more effectively reduced *S. aureus* than *S. epidermidis* single biofilms (37°C). Moreover, the two strains showed different temperature dependencies (37°C vs. 32°C). The advantage of *S*. *epidermidis* in mixed culture on the Gel‐Alg substrate was reversed under RL + VAN treatment.

Despite being limited to two species only, our data underlines the importance of culturing conditions for eventually studying the skin microbiome (Part A). Furthermore, our metagenomic analysis of Acne inversa smears (Part B) identified variations in bacterial composition between healthy and affected body regions, indicating that alterations in the microbiome should be considered when conducting in vitro analyses. The concept can be further expanded by the addition of more species and might have the potential to build a simplified platform for microbiome research. Also, the minimalistic gel substrate can possibly be improved toward representing certain skin components as a nutrition source for the bacterial communities. Insights obtained from such models of variable complexity may be valuable to study anti‐infective drugs in microbial communities and to develop improved therapeutic modalities for skin infections.

## 4 MATERIALS AND METHODS

### 4.1 Part A: Bacterial proliferation and susceptibity in skin models

#### 4.1.1 Cultivation of laboratory staphylococci strains

For the part of this work focusing on bacterial cultivation in different models, *S. aureus*, strain Newman (ATCC 25904) and *S. epidermidis* (ATCC 35984) were used. *S. aureus* was cultured in BHI broth and agar (Becton Dickinson, USA) while for *S. epidermidis* tryptic soy broth and agar (Becton Dickinson, USA), supplemented with yeast extract (Becton Dickinson, USA) (TSB‐YE) was used. Overnight cultures were started by inoculating one colony from an agar plate into an Erlenmeyer flask with 20 mL of the respective nutrient broth and shaking for approximately 16 h at 180 rpm and 37°C.

Biofilm formation and viability were first investigated via cultivation at 37°C and 32°C in broth and counting of colony‐forming units after 1, 3, and 6 days (Figure S2).

#### 4.1.2 Skin substrate Gel–Alg

The composition for the skin substrate was adapted from Quan et al. using gelatin, HA and CS. To increase scaffold strength and stability, it was further extended with alginate. First, alginate (FMC BioPolymer, UK) was weighed and put under UV light for 1 h before being dissolved in the respective amount of milliQ water (Milli‐QVR, Advantage A10, Merck Millipore, Germany) to achieve 8% (w/v). This step was performed using an autoclaved Schott flask and magnetic stirrer. Alginate solution was stirred overnight at 50°C–60°C. The next day 10% (w/v) gelatin (Type A, Gelita, Germany) was prepared in milliQ water and dissolved for 1 h at 50°C–60°C. In parallel, 0.1% (w/v) HA (Dagmar Köhler, Germany) and 0.2% (w/v) CS (VWR International, USA) were prepared and subsequently added to the solubilized gelatin to achieve a final concentration of 5, 0.1 and 0.05% (w/v) for Gel, HA and CS respectively. After further incubation at 50°C–60°C for 30 min, the mixture was sterile filtered (Minisart®, Sartorius Stedium, pore size 0.2 μm, Germany) and added to alginate in a 1:1 ratio under the sterile hood. After stirring another 30 min at 50°C–60°C, the substrate Gel‐Alg was ready to use. It can be stored at 4°C and heated at 50°C–60°C before using.

Since the Gel‐Alg substrate behaves more like a liquid, dissipating energy rather than storing it elastically (Figure S3), crosslinking with CaCl_2_ is required to provide a rather solid substrate for bacterial growth (Figure S4). For working in 48 well plates (greiner bio‐one, Germany), 100 μL of sterile filtered 100 mM CaCl_2_ (VWR International, USA) were therefore added and circa 500 μL of Gel‐Alg were transferred using a sterile syringe (Braun, Germany). The plates were closed with parafilm and left overnight at room temperature (RT). The next day, 100 μL of 100 mM sterile CaCl_2_ were added and after 1 h incubation at RT, the surface was washed with 300 μL sterile PBS (1× PBS, pH 7.4, Sigma‐Aldrich, Germany). The plates were left to dry under the sterile hood for about 30 min before inoculation with bacteria.

#### 4.1.3 Reconstructed human epidermis (RHE—EpiDerm™, MatTek)

The antibiotic‐free EpiDerm™ tissues (MatTek Europe, Slovak Republic) were handled according to the manufacturer's instruction. Upon arrival, EpiDerm™ tissues were transferred with sterile tweezers to a six‐well plate with 0.9 mL EPI‐MM ABF (MatTek Europe, Slovak Republic) and incubated for 1 h at 37°C and 5% CO_2_. Then the medium was exchanged, and the tissues were further incubated overnight prior to experiments with bacteria.

#### 4.1.4 Ex vivo skin

Native human skin was obtained from patients undergoing surgery (abdominoplasty, Kreiskrankenhaus St. Ingbert GmbH) according to the protocol approved by the Ethics Commission of the Chamber of Medicine Doctors of the Saarland (file number 116/20).

After removing the fat tissue with a sterile scalpel (Swann Morton, UK), the tissue was frozen at −20°C. One day prior to inoculation with bacteria, the skin was thawed at RT and an 11 mm whole punch (Gedore Henkellocheisen, Conrad Electronic, Germany) was used for biopsies. These were transferred to 24 well plates and 500 μL DMEM, supplemented with 10% FCS, 10% (v/v) Amphotericin B, and 10% (v/v) Pen/Strep (all Gibco, UK). After incubation for 4 h at 37°C and 5% CO_2_, the media was aspirated and a washing step with sterile PBS was performed. Then, 500 μL of the respective medium without Pen/Strep was added and tissues were incubated overnight under the same conditions. Susceptibility toward the different media as well as Pen/Strep and Amphotericin B are depicted in Figures S5 and S6.

#### 4.1.5 Biofilm cultivation in different models

Gel_HA_CS_‐Alg substrate, RHE, and human skin as well as respective overnight cultures were prepared as previously described. For broth cultures, 48 well plates were filled with 500 μL of the respective nutrient broth.

For *S. aureus*, 30 μL of bacteria were added to 10 mL BHI and the optical density (OD) was measured at 600 nm using BHI as blank. The procedure was similar for *S. epidermidis* using 700 μL bacteria and 10 mL TSB‐YE instead. The volume of diluted bacteria was calculated to reach an OD of 0.001 for *S. aureus* and 0.01 for *S. epidermidis* in 500 μL, corresponding to approximately 10^6^ log_10_(cfu/mL). Drops of 10–20 μL diluted bacteria were then added to the respective broth, Gel‐Alg or RHE samples. For skin samples, a higher volume of bacterial overnight cultures was used for dilutions and OD measurements to achieve the same bacterial count in drops of approximately 2 μL. Finally, cultures were incubated for 24 h at 37°C.

#### 4.1.6 Viability via colony‐forming units

To determine the number of viable bacteria, each sample (broth, Gel‐Alg, skin) was transferred to an Eppendorf tube and 1 mL PBS was added. For EpiDerm™ tissues, 100 μL PBS was added apically and after carefully pipetting transferred to an Eppendorf tube. This step was repeated and then tissues were flipped and the membrane, including the tissues, was cut out with a sterile scalpel. After turning it upside‐down again, 800 μL PBS was added. The plastic construct of the model was discarded, and the PBS was added to the same Eppendorf tube. Finally, the membrane with the tissue was transferred to the same tube using a sterile tweezer.

After vortexing for 10–20 min, 20 μL samples were used for serial dilution in 0.05% (v/v) Tween80 (MP Biomedicals, USA) in PBS and three drops of 20 μL of the respective dilutions were added on BHI or TSB‐YE agar plates. Plates were incubated overnight at 30°C–32°C and colonies were counted the next day to calculate cfu/mL.

#### 4.1.7 Metabolic activity via Presto Blue staining

A qualitative comparison of metabolic activity in the different models was performed via staining with Presto Blue (Invitrogen, USA). Samples were prepared as described above for cfu counting, including either a blank control of the system without bacteria or bacteria‐free PBS. One hundred fifty microliter of each sample were transferred to a 96 well plate, and 10% (v/v) Presto Blue was added. After incubation with slight shaking (300 rpm), absorbance was measured at 570 and 600 nm at several time points (5–90 min) using a plate reader (Multiscan Go, Thermo Scientific, Germany). The results are shown via the difference of OD (=OD_DIFF_) of both wavelengths.

#### 4.1.8 PMA treatment and viability qPCR

To differentiate between the two bacterial species in a co‐culture, qPCR was performed using commercial strain specific primers and TaqMan probes (PCR_max_® Human Microbial Pathogen Detection Kits for qPCR, Cole Parmer, USA). Biofilm cultures were prepared as described above with slight modifications. For broth cultures, a 1:1 mixture of BHI and TSB‐YE was used. Furthermore, prior to inoculation, the respective amount calculated for start OD/CFU of both bacteria was mixed in an Eppendorf tube and subsequently added to the respective models to achieve equal distribution of both strains in the droplets.

#### 4.1.9 Sample preparation and PMA treatment

After biofilm formation (24 h at 37°C), the bacterial cultures (broth, Gel‐Alg and RHE) were prepared as described for cfu counting and vortexed for 15–20 min. Then, 400 μL of each sample was pipetted to a new 1.5 mL Eppendorf tube and 1 μL of PMA_xx_ (100 μM stock, 1:1 mixed with fresh milliQ water, Biotium, USA) was added in order to exclude quantification of genomic DNA of non‐viable cells. The samples were incubated shaking for 10 min in the dark and subsequently exposed to light for 15 min via the PMA‐Lite™ LED Photolysis Device (Biotium, USA). A validation for PMA treatment included PMA‐treated and untreated live and dead controls and was performed according to the manufacturer's guidelines (Table S1).

#### 4.1.10 DNA isolation

PMA treated samples were centrifuged for 10 min at 5000 g and the pellet was resuspended in 180 μL lysozyme (20 mg/mL [VWR International, USA] in Tris–HCl [Sigma‐Aldrich, Germany] buffer pH 8 with 2 mM EDTA [Roth, Germany] and 1.2% (v/v) Triton X [Sigma‐Aldrich, Germany]). After incubation of at least 30 min at 37°C, the cells were lysed with lysis buffer and Proteinase K (both from QIAmp DNA Mini Kit, Qiagen, Netherlands) with an incubation of 30 min at 56°C and subsequently for 15 min at 95°C (Thermomixer Comfort, Eppendorf, Germany). Four microliter of an internal extraction control DNA (PCR_max_® kits) as well as 200 μL Ethanol were added and DNA was extracted using the QIAmp DNA Mini Kit (Qiagen, Germany) according to the manufacturer's instructions.

#### 4.1.11 Viability qPCR

DNA samples were stored at 4°C prior to PCR measurements. The mastermixes including strain‐specific primers and probes for *S. aureus* and *S. epidermidis* (PCR_max_® kits) were prepared as described in the manufacturer's instructions and 7.5 μL were pipetted to a 96 well half‐skirted PCR plate (VWR International, USA), leaving the wells of the outer rim empty. Subsequently, 2.5 μL of the respective DNA sample were added and qPCR was run with a thermal cycler (CFX96 Real‐Time System with C1000 Thermal Cycler, BioRad, Germany) using the recommended protocol for PCR_max_® kits.

#### 4.1.12 Crystal violet assay

To investigate the biomass reduction by RLs (Sigma‐Aldrich, Germany), a serial dilution of 20 mg/mL in PBS was performed in 200 μL PBS in a 96 well plate. Biofilm of *S. aureus* and *S. epidermidis* was cultivated as described above using 200 μL of the respective broth in 96 well plates instead. Prior to treatment, the medium was aspirated and 200 μL of the respective RL dilutions were transferred to the biofilms; PBS was used as a control. Plates were incubated for 2 h at 37°C, the medium was aspirated, and the biofilms were fixed with 200 μL methanol for 15 min. Methanol was removed by inverting the plate and 200 μL 0.5% (w/v) CV (Merck, Germany) in 20% methanol was added. After 15 min incubation in the dark, CV was removed by inverting the plate and three washing steps with water were performed. CV was subsequently dissolved by addition of 30% acetic acid and after 5–10 min incubation OD was measured at 630 nm via a plate reader (Multiscan Go, Thermo Scientific, Germany). To calculate the relative biomass (%) values were normalized to the untreated control.

#### 4.1.13 Biofilm staining and confocal microscopy

*S. aureus* and *S. epidermidis* biofilms were grown in broth in 48 well plates as described before. After 24 h, the medium was aspirated and 500 μL 5 mg/mL RLs were added. As an untreated control, 500 μL PBS were used instead. After 2 h incubation at 37°C, medium was carefully removed, and samples were fixed with 500 μL methanol for 15 min. Then, 400 μL Film Tracer SYPRO ruby (Invitrogen, USA) were added to stain the biofilm matrix, and samples were incubated in the dark for 30 min. After washing with water, imaging was performed with a DMi8 inverted microscope and a TCS SP8 confocal laser scanning unit with an AOBS beam splitter and HyD detector (Leica, Germany) as well as a Fluotar VISIR 25×/0.95 water objective. For excitation, an argon laser (30%) was used at 488 nm with 15% intensity, and emission was detected at 610 nm.

#### 4.1.14 Susceptibility of rhamnolipid and vancomycin co‐treatment

First, the efficacy of the co‐treatment was tested in the broth condition only; however, including the relevant skin temperature of 32°C for biofilm cultivation and incubation after treatment. RL and vancomycin (Alpha Aesar, Germany) stocks were prepared in PBS and sterile filtered.

*S. aureus* and *S. epidermidis* biofilms were prepared in 96 well plates with a volume of 200 μL as described before. After 24 h cultivation at 37°C or 32°C, the medium was carefully aspirated with a pipette and 100 μL of PBS, 0.5 or 5 mg/mL RLs were added. The cultures were incubated for 2 h at the respective temperature. 4 mg/mL vancomycin was prepared in PBS and 1:1 mixed with the respective nutrient broth. 100 μL was then added to achieve a final concentration of 1 mg/mL. Plates were further incubated for 24 h, and serial dilution as well as cfu plating and counting was performed for quantification of viable bacteria.

#### 4.1.15 Co‐treatment in different models

To test the treatment efficacy in different models, the cultures (broth, Gel‐Alg and RHE) were prepared as previously described with incubation at 37°C only. After 24 h, 5 mg/mL RLs were added since the higher dose showed better efficacy. For broth cultures in 48 well plates, medium was aspirated and 250 μL RLs were added; PBS was used as a control. For Gel‐Alg and RHE, only 50 μL of RLs or PBS were pipetted on top of the cultures. After 2 h incubation, 250 μL (broth) or 50 μL (Gel‐Alg and RHE) of 2 mg/mL vancomycin or PBS were added respectively. Cultures were incubated for an additional 24 h and viability was again assessed via cfu counting. Results are shown as Δlog_10_(cfu/mL):

$${\text{Δlog}}_{10}{\left(\mathrm{cfu}/\mathrm{mL}\right)}_{\text{treatment}}={\mathrm{cfu}}_{\text{treated}}-{\mathrm{cfu}}_{\text{untreated}}$$

#### 4.1.16 Co‐treatment of bacterial co‐cultures

For treatment of bacterial co‐cultures a lower RL concentration of 0.5 mg/mL was chosen due to possible membrane damage and consequently inconclusive results via PMA staining and qPCR analysis (data not shown).

Bacterial co‐cultures in broth and on Gel‐Alg were prepared and treated with RLs and vancomycin as stated, followed by quantification via viability qPCR. To compare treatment efficacy based on ct values, the following calculations were used:

$${\mathrm{\Delta ct}}_{\text{treatment}}={\mathrm{ct}}_{\text{untreated}}-{\mathrm{ct}}_{\text{treated}}$$

$${\text{ΔΔct}}_{\text{treatment}}={\mathrm{\Delta ct}}_{\text{treatment}-\text{single}}-{\mathrm{\Delta ct}}_{\text{treatment}-\text{mixed}}$$

#### 4.1.17 Statistical analysis

All experiments were performed three times with triplicates each (*N* = 3 experiments with *n* = 9 samples). Statistical analysis was performed using Microsoft excel and paired *t* test or one way ANOVA followed by the Tukey test as stated in the respective figure caption. Statistical difference was considered significant for *p* < 0.05 and is indicated by asterisks (*) or hashes (#). Significance levels are depicted in Data S1 for Figure 2 (Figure S1, respectively) and Figure 5 (Tables S2 and S3).

### 4.2 Part B: Metagenomic analysis of Acne inversa smears

#### 4.2.1 Ethics statement

A written informed consent was obtained from all participants prior to sample collection. The study was approved by the Ethics Commission of the Chamber of Medicine Doctors of the Saarland (file number 131/20) and samples from *N* = 18 patients were used.

#### 4.2.2 Culturing of bacteria from native samples and subsequent MALDI‐TOF analysis

Samples of 18 patients were used to investigate the microbiome of Acne inversa patients. From each individual, two smears were used: one from diseased body regions and one from unaffected body regions (forehead or arm).

To ensure comprehensive microbiological analysis, all native samples underwent streaking on two distinct agar plates: Tryptic Soy Agar with 5% sheep blood (TSA) and Columbia (Co) agar plates (ThermoFisher Scientific, Wilmington, DE). Incubation of TSA plates transpired at 35.6°C with 5% CO2, lasting between 18 and 24 h. For the cultivation of anaerobic bacteria, Co agar plates were utilized and incubated in an anaerobic environment for a minimum of 48 h.

Following bacterial culturing on diverse agar plates, colonies were transferred to the MALDI‐TOF target plate and overlaid with 1 μL of α‐cyano‐4‐hydroxycinnamic acid (CHCA) matrix solution (Bruker Daltonics). This matrix solution consists of saturated CHCA dissolved in 50% (v/v) acetonitrile, 2.5% (v/v) trifluoroacetic acid, and 47.5% (v/v) LC–MS grade water. After air‐drying at room temperature, the plate was introduced into the Microflex LT Mass Spectrometer (Bruker Daltonics) for MALDI‐TOF MS analysis. Utilizing the AutoXecute algorithm in FlexControl software (v3.4; Bruker Daltonics), each spot underwent 240 laser shots at six random positions, generating protein mass profiles in linear positive ion mode. The parameters included a laser frequency of 60 Hz, a high voltage of 20 kV, and a pulsed ion extraction of 180 ns. Mass charge ratio range (m/z) was measured from 2 to 20 kDa. Bacterial species identification was conducted using the MALDI BioTyper software. Scores exceeding 2.0 indicated precise identification, scores between 1.7 and 1.99 suggested possible species identification, and identification scores below 1.7 were regarded as unsuccessful identification.^50^, ^51^

#### 4.2.3 DNA extraction

All native skin swabs medium (eSwabs, Copan Diagnostics, USA in respective transport medium AMIES) were exposed to rigorous vortexting for 3 min to remove bacterial mass from the nylon‐flocked swab into the provided AMIES medium. Next, 1 mL of AMIES medium, containing the bacterial cells, was subjected to nucleic acid extraction using the Qiagen QiAamp DNA Microbiome Kit (Qiagen, Germany). Briefly, host DNA was depleted by adding Buffer AHL in a 1:2 ratio and subsequently degrade human DNA using Benzoase. Next, bacterial cells were lysed by combining mechanical and chemical lysis using the lysis buffer provided by Qiagen, and a mechanical lysis protocol was carried out using the FastPrep‐24 5G Instrument (FisherScientific, Germany) set to 6.5 m/s for 45 s, two times with a 5 min storage on ice between each lysis step. Released bacterial DNA was then purified using the provided UCP columns, containing a silica membrane.

DNA was eluted with 50 μL buffer AVE. Concentration and purity of the extract nucleic acid were assessed using the NanoDrop 2000/2000c (ThermoFisher, Germany).

#### 4.2.4 Sequencing

The complete genomic DNA was dispatched to Novogene Company Limited, located in Cambridge, UK, for the purpose of library preparation and subsequent sequencing. In summary, the extracted nucleic acids underwent metagenomic library preparation and were subsequently subjected to short‐read sequencing via Illumina Sequencing PE150 (HiSeq). In each case, a total of 3 gigabases (Gb) of reads were generated per sample.

#### 4.2.5 Metagenomic data analysis

Quality controlled and decontaminated samples mentioned in was taken from Rehner et al.^45^ Utilizing MetaPhlAn3 55 (version: 3.0.13; command line arguments: “‐t rel_ab_w_read_stats–unknown_estimation–add_viruses”), profiling of quality‐controlled samples was performed on the mpa_v30_CHOCOPhlAn_201901 database. The relative counts obtained were adjusted to absolute counts, taking into consideration both the number of reads and the removal of virus counts. Diseased specimens were kept. If forehead and arm microbiome were available for a patient, the sample with fewer reads was discarded. Ordination analysis was performed on MinHash distances computed with Mash (v: 2.3; cla: “sketch ‐S 23 ‐k 31 ‐s 5000 ‐r ‐m 2”). Embeddings were computed with the UMAP package 60 (v: 0.2.8)./Ordination analysis used Bray Curtis distance measure on species abundances. For visualization non‐metric distance scaling was performed. PERMANOVA test was computed comparing healthy and diseased specimen using the vegan package in R (v:2.6.2). As an alpha diversity measure, we used Shannon diversity. Statistical significance was assessed using an unpaired Wilcoxon rank sum test. Differential abundance analysis was performed with ANCOMBC (v:1.6.2). For sample assembly, SPAdes 62 (version: 3.15.4; command line arguments: “‐‐meta”) was employed.

## AUTHOR CONTRIBUTIONS

Conceptualization: Sarah Frisch, Brigitta Loretz, Thomas Vogt and Claus‐Michael Lehr. Data curation: Sarah Frisch, Samy Aliyazdi, Jacqueline Rehner, Georges Schmartz, and Caroline Gevaerd. Supervision: Lorenz Latta, Sören L. Becker, Andreas Keller, Ulrich F. Schaefer, Thomas Vogt, Brigitta Loretz and Claus‐Michael Lehr. Writing original draft: Sarah Frisch. All authors have read and agreed to the published version of the manuscript. The manuscript was written through the contributions of all authors.

## CONFLICT OF INTEREST STATEMENT

Georges Schmartz and Andreas Keller are co‐founders of MooH GmbH, a company developing metagenomic‐based oral health tests.

## Supporting information


**Figure S1:** Growth and metabolic activity in different conditions: broth, skin substrate Gel‐Alg, reconstructed human epidermis (RHE, EpiDerm™, MatTek) and ex vivo skin. Growth via log10(cfu/mL) of *S. aureus* (A) and *S. epidermidis* (B) single biofilms. One‐Way ANOVA revealed significant difference of ex vivo skin to all other conditions tested. Metabolic activity via Presto Blue staining and OD_DIFF_ of *S. aureus* (C) and *S. epidermidis* (D) single biofilms. All conditions showed statistically significant difference as tested via one‐way ANOVA with *p < 0.05, **p < 0.01, ***p < 0.005. *N* = 3 experiments with *n* = 9 samples.
**Figure S2:** Growth via log10(cfu/mL) of *S. aureus* (A) and *S. epidermidis* (B) single biofilms after 1, 3 and 6 days at 37°C and 32°C. *N* = 3 experiments with *n* = 9 samples.
**Figure S3:** Rheological characterization of Gel‐Alg substrate by frequency. *N* = 3 experiments with *n* = 3 samples.
**Figure S4:** Confocal imaging of *S. aureus* GFP in broth and on Gel‐Alg substrate with z‐stacks of 2.2 mm. Height difference reflects the substrate thickness, and the images confirm that bacteria grow on the surface of the Gel‐Alg substrate rather than inside.
**Figure S5:** Susceptibility of *S. aureus* towards skin pre‐medium (with both, antibiotics and amphotericin), the cultivation medium (free of anti‐infectives) as well as Pen‐Strep and Amphotericin in PBS instead of medium, but with similar concentrations. *N* = 3 with *n* = 9.
**Figure S6:** Susceptibility of *S. epidermidis* toward skin pre‐medium (with both, antibiotics and amphotericin), the cultivation medium (free of anti‐infectives) as well as Pen‐Strep and Amphotericin in PBS instead of medium, but with similar concentrations. *N* = 3 with *n* = 9.
**Table S1:** PMA validation for *S. aureus* and *S. epidermidis* using live and dead control. The dCT of PMA‐treated and untreated samples is shown and fold reduction and as well as viability were compared according to the manufacturer's specification. Viability was within the range of validation. *N* = 3 with *n* = 6.
**Table S2:** Rhamnolipid (no −, low +, and high ++ concentration, 0.5 and 5 mg/mL respectively) and Vancomycin (1 mg/mL) efficacy via log10(cfu/mL) at 37°C and 32°C for *S. aureus* and *S. epidermidis* single biofilms. The Δlog10(cfu/mL) is displayed for treatment including significance via One‐Way ANOVA. *N* = 3 experiments with n = 9 samples.
**Table S3:** Rhamnolipid (no −, low +, and high ++ concentration, 0.5 and 5 mg/mL respectively) and Vancomycin (1 mg/mL) efficacy via log10(cfu/mL) at 37°C and 32°C for *S. aureus* and *S. epidermidis* single biofilms. The Δlog10(cfu/mL) is displayed for difference in temperature via paired t‐test. *N* = 3 experiments with n = 9 samples.

## Data Availability

The metagenomic sequencing data after removing ambient human DNA generated in this study has been deposited in the Sequencing Read Archive under the accession code PRJNA1057503. Further data that support the findings of this study are available from the corresponding author upon reasonable request.
