# Supplementary material for: Staphylococcal proliferation on skin models to investigate novel anti‐infective treatments against dysbiosis
Source: Bioeng Transl Med. 2026 Mar 26;11(3):e70124. doi: 10.1002/btm2.70124 (PMC13247412; doi:10.1002/btm2.70124)
Supplement: Supplementary file 1 — Figure S1: Growth and metabolic activity in different conditions: broth, skin substrate Gel‐Alg, reconstructed human epidermis (RHE, EpiDerm™, MatTek) and ex vivo skin. Growth via log10(cfu/mL) of S. aureus (A) and S. epidermidis (B) single biofilms. One‐Way ANOVA revealed significant difference of ex vivo skin to all other conditions tested. Metabolic activity via Presto Blue staining and ODDIFF of S. aureus (C) and S. epidermidis (D) single biofilms. All conditions showed statistically significant difference as tested via one‐way ANOVA with *p < 0.05, **p < 0.01, ***p < 0.005. N = 3 experiments with n = 9 samples. Figure S2: Growth via log10(cfu/mL) of S. aureus (A) and S. epidermidis (B) single biofilms after 1, 3 and 6 days at 37°C and 32°C. N = 3 experiments with n = 9 samples. Figure S3: Rheological characterization of Gel‐Alg substrate by frequency. N = 3 experiments with n = 3 samples. Figure S4: Confocal imaging of S. aureus GFP in broth and on Gel‐Alg substrate with z‐stacks of 2.2 mm. Height difference reflects the substrate thickness, and the images confirm that bacteria grow on the surface of the Gel‐Alg substrate rather than inside. Figure S5: Susceptibility of S. aureus towards skin pre‐medium (with both, antibiotics and amphotericin), the cultivation medium (free of anti‐infectives) as well as Pen‐Strep and Amphotericin in PBS instead of medium, but with similar concentrations. N = 3 with n = 9. Figure S6: Susceptibility of S. epidermidis toward skin pre‐medium (with both, antibiotics and amphotericin), the cultivation medium (free of anti‐infectives) as well as Pen‐Strep and Amphotericin in PBS instead of medium, but with similar concentrations. N = 3 with n = 9. Table S1: PMA validation for S. aureus and S. epidermidis using live and dead control. The dCT of PMA‐treated and untreated samples is shown and fold reduction and as well as viability were compared according to the manufacturer's specification. Viability was within the range of validation. [file BTM2-11-e70124-s001.docx]

**Supporting Information**

Staphylococcal proliferation on skin models to investigate novel anti-infective treatments against dysbiosis

Sarah Frisch^1,2^, Samy Aliyazdi^1,2^, Jacqueline Rehner^2,3^, Georges Schmartz^2,4^, Caroline Gevaerd^5^, Lorenz Latta^1^, Barbara Veldung^6^, Sören L. Becker^2,3,7^, Andreas Keller^2,4,7^, Ulrich F. Schaefer^2^, Brigitta Loretz^1,7^, Thomas Vogt^5^, Claus-Michael Lehr^1,2,7^*

# ^1^Helmholtz-Institute for Pharmaceutical Research Saarland, Helmholtz Center for Infection Research, Saarbrücken, Germany; ^2^Saarland University, Saarbrücken, Germany; ^3^Institute of Medical Microbiology and Hygiene, Saarland University, 66421, Homburg, Germany; ^4^Clinical Bioinformatics, Saarland University, 66123, Saarbrücken, Germany; ^5^Clinic for Dermatology, Venereology, and Allergology, 66421, Homburg, Germany; ^6^Specialist in Plastic and Aesthetic Surgery, Saarbrücken, Germany ^7^PharmaScienceHub, Saarland University, Campus A2 3, 66123 Saarbrücken

# **Statistical Analysis (Figure 2)**

# Growth and metabolic activity of *Staphylococcus aureus* and *Staphylococcus epidermidis* were investigated in four different models as described in the manuscript (Experimental section - Biofilm Cultivation in different models). Results are shown in Figure 3 but do not depict results of statistical analysis in detail. Hence, below Figure S1 depicts statistically significant differences as asterisks (*p<0.05, **p<0.01, ***p<0.005).


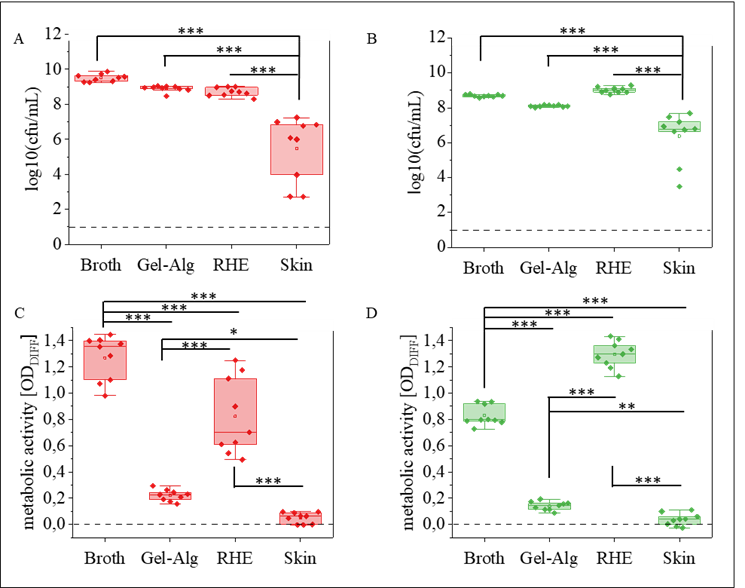


Figure S1: Growth and metabolic activity in different conditions: broth, skin substrate Gel-Alg, reconstructed human epidermis (RHE, EpiDerm^TM^, MatTek) and ex vivo skin. Growth via log10(cfu/mL) of S. aureus (A) and S. epidermidis (B) single biofilms. One-Way ANOVA revealed significant difference of ex vivo skin to all other conditions tested. Metabolic activity via Presto Blue staining and OD_DIFF_ of S. aureus (C) and S. epidermidis (D) single biofilms. All conditions showed statistically significant difference as tested via One-Way ANOVA with *p<0.05, **p<0.01, ***p<0.005. N=3 experiments with n=9 samples

# **Growth Curve**

Both strains, *Staphylococcus aureus* and *Staphylococcus epidermidis*, were cultivated as described in the manuscript (Experimental Section – Cultivation of laboratory staphylococcal strains /Biofilm cultivation in different models) for nutrient broth. In this set-up, both temperatures of 32°C and 37°C were tested with a read-out via counting of colony-forming units after 1, 3 and 6 days. The experiment revealed similar growth for S. aureus over time while S. epidermidis only achieved same values after one day culture. For 3 and 6 days, a decrease was noted for cultivation at 37 °C but not at 32 °C.

Figure S2: Growth via log10(cfu/mL) of S. aureus (A) and S. epidermidis (B) single biofilms after 1, 3 and 6 days at 37°C and 32°C. N=3 experiments with n=9 samples

# **Gel-Alg Rheological Characterization**

To investigate bulk rheology of the Gel-Alg substrate a Discovery HR-2 Hybrid Rheometer (Waters/TA Instruments, USA) with Smart Swap™ Peltier Plate geometry (diameter: 20 mm, cone angle: 1 °) was used. 100 μL of Gel-Alg (not crosslinked) were placed between the measuring units, frequency sweep experiments in a range of 0.1 to 100 rad/s were performed at 25 °C to determine the elastic (G') and viscous (G'') moduli.

The results in Figure S3 showed that the loss modulus (G'') is higher than the storage modulus (G') with increasing frequency. Only at very low frequency G' is higher than G''. Therefore, the material behaves more like a liquid, dissipating energy rather than storing it elastically and crosslinking with CaCl_2_ is required to provide a rather solid substrate for bacterial growth.

Figure S3: Rheological characterization of Gel-Alg substrate by frequency. N=3 experiments with n= 3 samples

**Gel-Alg CLSM with *S. aureus* GFP**

For visualising bacteria on the Gel-Alg substrate *S. aureus* Newman GFP (ATCC 25904-pCtuf-gfp) was used. Overnight cultures were prepared by inoculating a colony from BHI-agar plates with chloramphenicol and shaking in 20 mL BHI medium with chloramphenicol at 37 °C. Then, bacteria were cultivated in BHI and on Gel-Alg as described in the manuscript using a 4-well chambered coverglass system (Thermo Scientific, USA) instead of well plates. After 24 h incubation, bacterial cultures of *S. aureus* GFP were investigated via confocal laser scanning microscopy (CLSM). Imaging was carried out using a DMi8 inverted microscope equipped with a TCS SP 8 confocal laser scanning system featuring an AOBS beam splitter and a HyD detector (Leica, Germany). For excitation, a 20% argon laser at 476 nm was employed, while emission was measured at 580 nm. Z-stack images covering a depth of 2.2 mm were obtained with a Fluotar VIZIR 24×/0.95 water immersion objective.

Figure S4 displays *S. aureus* GFP images in broth and on Gel-Alg substrate under similar conditions. The height difference reflects the substrate thickness, and the images confirm that bacteria grow on the surface of the Gel-Alg substrate rather than inside.

# **
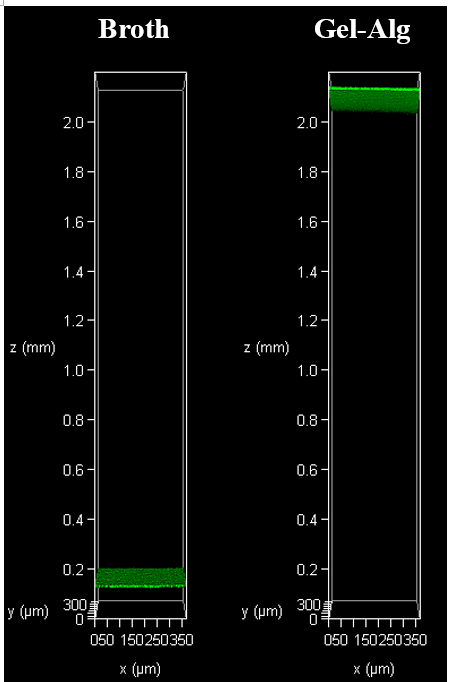
**

Figure S4: Confocal imaging of S. aureus GFP in broth and on Gel-Alg substrate with z-stacks of 2.2 mm. Height difference reflects the substrate thickness, and the images confirm that bacteria grow on the surface of the Gel-Alg substrate rather than inside.

# **MIC Skin medium**

In order to decontaminate the skin samples, a specific medium is used which contains Penicillin, Streptomycin as well as Amphotericin. The concentrations are reduced stepwise, and the final medium used for bacterial cultivation was free of anti-infectives. Still, a check for susceptibility of our laboratory staphylococcal strains was performed to determine the critical concentrations for each component (Figure S2 for *S. aureus* and Figure S3 for *S. epidermidis*). Therefore, a MIC via OD measurement was performed using four conditions: The pre-medium (with both, antibiotics and amphotericin), the cultivation medium (free of anti-infectives) as well as Pen-Strep and Amphotericin in PBS instead of medium, but with similar concentrations. The data showed that *S. aureus* is highly sensitive towards Pen-Strep and consequently also the pre-medium, with the lowest dilution tested still inhibiting bacterial growth. *S. epidermidis*, on the other hand, was only inhibited using the two highest doses of 500 and 1000 µg/mL. Amphotericin did not show any inhibition even at the highest dose of 25 µg/mL for both tested strains. As expected, also the anti-infective-free cultivation medium did not reveal any negative impact on bacterial growth in this set-up.

Figure S5: Susceptibility of S. aureus towards skin pre-medium (with both, antibiotics and amphotericin), the cultivation medium (free of anti-infectives) as well as Pen-Strep and Amphotericin in PBS instead of medium, but with similar concentrations. N=3 with n=9

Figure S6: Susceptibility of S. epidermidis towards skin pre-medium (with both, antibiotics and amphotericin), the cultivation medium (free of anti-infectives) as well as Pen-Strep and Amphotericin in PBS instead of medium, but with similar concentrations. N=3 with n=9

# **PMA validation**

PMA treatment was used as an indicator for viability when determining ct values via qPCR. To validate this technique four different conditions were tested: (i) live control, PMA-treated, (ii) live control, untreated, (iii) dead control, PMA-treated, and (iv) dead control, untreated – all conditions for both laboratory strains. Bacteria were cultivated as described in the manuscript for 24 h in nutrient broth ((Experimental Section – Cultivation of laboratory staphylococcal strains /Biofilm cultivation in different models). Samples were transferred to Eppendorf tubes and the dead control samples were heat-killed at 95 °C for 5 min. Then, PMA treatment and qPCR were performed accordingly (Experimental Section – PMA treatment and viability qPCR). Viability values was based on dCT and fold reduced, which were calculated using the formulas:

dCt = Ct_PMA-treated_ - Ct_untreated_

Fold reduced by PMA = ^dCt^

% viable = 100 / Fold reduced

Values were compared to the manufacturer`s specification and were within the expected range for live (dCt close to 0 +/- 1) and dead control (dCt >4). Data is shown in Table S1.

Table S1: PMA validation for S. aureus and S. epidermidis using live and dead control. The dCT of PMA-treated and untreated samples is shown and fold reduction and as well as viability were compared according to the manufacturer`s specification. Viability was within the range of validation. N=3 with n=6

|  | Condition | dCt | Fold reduced | Viability |
| --- | --- | --- | --- | --- |
| *S. aureus* | Live control | -0.1 | 0.9 | 105.6 |
|  | Dead control | 7.6 | 190.7 | 0.5 |
| *S. epidermidis* | Live control | 0.9 | 1.9 | 53.9 |
|  | Dead control | 8.0 | 262.0 | 0.4 |

# **Co-treatment of Rhamnolipid and Vancomycin**

Susceptibility of Rhamnolipid and Vancomycin were tested in different conditions as depicted in Figure 5 of the main manuscript. We only added some hashes and asterisks that underline the main statements of this experiment. However, statistically significant differences were observed for several conditions, which are shown below in Table S2 (difference between treatment groups) and Table S3 (difference in temperature).

Table S 2: Rhamnolipid (no -, low +, and high ++ concentration, 0.5 and 5 mg/mL respectively) and Vancomycin (1 mg/mL) efficacy via log10(cfu/mL) at 37°C and 32°C for S. aureus and S. epidermidis single biofilms. The Δlog10(cfu/mL) is displayed for treatment including significance via One-Way ANOVA. N=3 experiments with n=9 samples

| untreated (RL-VAN-) vs. | | RL-VAN+ | RL+VAN- | RL+VAN+ | RL++VAN- | RL++VAN+ |
| --- | --- | --- | --- | --- | --- | --- |
| *S. aureus* | 37°C | 0.48 * | 0.55 ** | 2.23 *** | 1.60 *** | 7.91 *** |
|  | 32°C | 0.66 *** | 0.92 *** | 1.40 *** | 1.68 *** | 1.68 *** |
| *S. epidermidis* | 37°C | 0.07 ^ns^ | 0.34 *** | 1.55 *** | 0.55 *** | 4.47 *** |
|  | 32°C | 0.20 ^ns^ | 0.20 ^ns^ | 4.30 *** | 0.67 ** | 7.09 *** |

Table S 3: Rhamnolipid (no -, low +, and high ++ concentration, 0.5 and 5 mg/mL respectively) and Vancomycin (1 mg/mL) efficacy via log10(cfu/mL) at 37°C and 32°C for S. aureus and S. epidermidis single biofilms. The Δlog10(cfu/mL) is displayed for difference in temperature via paired t-test. N=3 experiments with n=9 samples

| 37°C vs 32°C | RL-VAN- | RL-VAN+ | RL+VAN- | RL+VAN+ | RL++VAN- | RL++VAN+ |
| --- | --- | --- | --- | --- | --- | --- |
| *S. aureus* | 0.27 ** | 0.09 * | 0.10 ^ns^ | 1.10 *** | 0.18 ** | 6.50 *** |
| *S. epidermidis* | 0.19 *** | 0.07 ^ns^ | 0.34 ** | 2.55 *** | 0.08 ns | 2.42 *** |
